# Self-rated health inequalities in the intersection of gender, social class and regional development in Spain: exploring contributions of material and psychosocial factors

**DOI:** 10.1186/s12939-020-01202-7

**Published:** 2020-06-05

**Authors:** Núria Pedrós Barnils, Eva Eurenius, Per E. Gustafsson

**Affiliations:** 1 Department of Physics, Polytechnic University of Catalonia, Barcelona, Spain; 2 Department of Epidemiology and Global Health, Umeå University, SE-901 87 Umeå, Sweden

## Abstract

**Background:**

Inequalities in health across social class, gender and regional context in Spain are well-known; however, there is a lack of research examining how these dimensions of inequality interact. This study explores self-rated health (SRH) inequalities across intersectional positions of gender, social class and region, and the contribution of material and psychosocial factors to these inequalities.

**Methods:**

Participants were drawn from the cross-sectional 2015 National Living Conditions Survey of Spanish residents aged 19–88 years (*N* = 27,215; 77% response rate). Eight intersectional positions were formed by combining dichotomous variables of gender, social class and regional development. Poisson regression was used to estimate intersectional inequalities in SRH as prevalence ratios, and the contributions of material and psychosocial factors.

**Results:**

Results showed both cumulative and heterogeneous inequalities within and across intersectional positions. Inequalities in the intersection of social class and regional development were best explained by the joint contributions of material and psychosocial factors, while gender inequalities within non-manual social class were better explained by material factors alone.

**Conclusions:**

The results illustrate the complexity of interacting inequalities in health and their underpinnings in Spain. Local and national policies taking this complexity into account are needed to broadly improve equity in health in Spain.

## Background

It is widely recognised that there are unacceptable social inequalities in health among different population groups, which potentially could be reduced through equity promoting policies and inclusive interventions [1]. These notions are embraced by The Commission to Reduce Social Inequalities in Health in Spain [2].

Social inequalities in health are however, a complex and pervasive phenomenon present across multiple dimensions. For example, gender inequalities are found in phenomena such as women having longer life expectancy than men, but poorer health when it comes to chronic illnesses, mental health or self-rated health (SRH) [3, 4]. Social class inequalities are widely studied [5, 6] showing that individuals in lower socioeconomic positions have higher risk of morbidity and earlier mortality than those in higher socioeconomic positions. Individuals’ health status can also differ depending on geographical contexts, as illustrated by regional inequalities within countries [7, 8].

Policies towards equity in health traditionally understand, describe and analyse such multiple social inequalities as separate and disentangled phenomena. However, more recent perspectives [9] acknowledge the interwoven nature of social inequalities, the influencing context, and the non-uniform effects this may have on population’s health. For example, health status may not equate to the simple sum of individual’s experiences of multiple disadvantages, as would be suggested from a so-called ‘additive approach’ [10]. The perspective of intersectionality, which takes into account such complexity, has therefore been advocated in public health research [11]. Diverse quantitative examples in population health research [11, 12] demonstrate how intersectional approaches taking combinations of disadvantages into account – a ‘multiplicative approach’- are promising for providing more precise evidence for designing interventions and strategies towards equity in health [9]. Following these recommendations, intersectional approaches have recently begun to be applied more broadly in quantitative equity in health research. Intersectionality has in this body of research been operationalized using a variety of specific methods [13–16], based on e.g. comparison of cross-classified social positions, estimation of interaction effects, or through random effects in multilevel models. While varying in their specific operationalization, these methods all have in common that they disclose population patterns of health in or across the intersections of multiple social positions, and are therefore all considered ‘multiplicative’ approaches in the present report, in accordance with the terminology of Bauer et al. (2014) [9].

Spain is a socio-democratic country divided in Autonomous Communities. Public policies in education, health and welfare are delivered by the Communities creating, in some cases, unfair disadvantages in terms of health and social welfare across them [17]. For example, Montiel [18] encountered indicators of mental health disparities across Autonomous Communities, and Nolasco [19] found that higher risk of death was associated to Spanish regions with higher levels of deprivation, especially among men. Gender and social class inequalities also affect the health status of the Spanish population. A report of the Spanish Government showed that only 55% of women in the lowest social class reported good health, compared to 85% of men in the highest social class [20]. Moreover, Aguilar-Palacio [21] found that individuals with low education level had a greater risk of poor SRH than those with high level. The largest risk of poor SRH among women was found in Andalucia, Canarias, Galicia and Murcia, illustrating interaction among different axes of inequality in shaping population patterns of health.

The Commission on Social Determinants of Health of the World Health Organization [22] proposed a framework whereby intermediary, structural social and economic determinants unequally affect health and wellbeing. Structural social and economic determinants are built upon the socioeconomic and political context and upon the individual social position. Intermediary determinants comprise material circumstances, psychosocial processes and behaviours and biological factors [23]. Many studies have assessed the intermediary determinants of health in relation to social positions of gender or social class [24, 25], but less so their contribution to intersectional inequalities in health. A Spanish example demonstrates contribution of intermediary determinants, including material factors, on the intersection between gender and social class and its effects on SRH [26]. Nevertheless, very little is known about the contribution of material and psychosocial factors on SRH in the intersection between gender, social class and region in Spain.

The aims of this study are therefore to (1) explore how intersectional social positions of social class, gender and region are reflected in population patters of SRH among Spanish adults, and (2) examine the contribution of intermediary social processes material and psychosocial factors to these inequalities in SRH. To illustrate the added value of using an intersectionality-informed multiplicative approach when it comes to population patterns in health, these questions will also be addressed from a conventional additive approach.. In the additive approach, gender, social class and regional inequalities in health will be approached separately and the health risks of groups in the intersections of multiple social positions will thus not be considered.

## Methods

### Study population, sampling and procedures

The study population (*N* = 27,215) comprised respondents of the 2015 annual national cross-sectional Living Conditions Survey ‘Encuesta de Condiciones de Vida’ [27], carried out by the National Institute of Statistics of Spain, Instituto Nacional de Estadística (INE). The survey is the source of the Spanish data that is part of the European Union Statistics on Income and Living Conditions (EU-SILC), one of the statistical operations that have been harmonised for EU countries and that is governed by European Union Statistical Office (Eurostat) of the European Commission. The Living Conditions Survey has been ongoing since 2004 and comprises four independent sub-samples, each of which is a four-year panel, and with the sample rotated in one of the panels each year. The 2015 survey corresponds to rotational group “3”. The survey procedures are described in detail in the technical reports by INE [27, 28] and in the European Commission Regulations describing definitions [29], fieldwork and imputation procedures [30], sampling and monitoring [31], target variables [32] and quality reports [33].

The target population for the 2015 survey comprised all non-institutionalised people resident in Spain aged 16 years old or older as of 31st December 2014 [27].The municipal population register was used to identify a stratified random sample, with an independent sample drawn within each Autonomous Community (self-governing region of Spain) as the INE is required to produce reliable data at this level of disaggregation as well as nationally. First, census sections, each consisting of about 400 addresses, were selected stratified for municipality size, with a selection probability proportional to the number of households within each census. Second, addresses were randomly sampled within each municipality, and personal interviews were held with all eligible household members. The sampling frame enables derivation of survey sampling weights, which were used in this analysis to aid generalization of the results to the target population.

Data collection was carried out through computer-assisted personal interviews with all participants in March – July 2015, with an average interview duration of 27 min. The content of the questionnaire is defined by the European Commission Regulations [32]. Training of interviewers was done at the provincial area level according to training manuals. If a personal interview was impracticable because the subject was temporarily absent or was unable to respond, a telephone interview or interview with another household member was conducted, and later the information was corroborated with the participant in question.

The INE ensures confidentiality during the data collection process and provides information on the use and confidentiality of the data to the respondents, and all participants give informed consent to the use of the data for research purposes. Quality assurance by the INE is based on the European Statistics Code of Practice made by Eurostat, and Eurostat also carries out independent review of the survey data before the results are published [27]. One of the weak points of the survey pointed out by Eurostat is the relatively high fraction of proxy interviews, the extension of which, however, has decreased since 2010 [27].

Complete public domain microdata files from the survey are disseminated free of charge by the INE in anonymized form and was for the present project retrieved from the official microdata download page of INE [34]. The overall individual response rate of the 2015 survey was 80.4% [35]. After excluding records with incomplete data on the study variables, a sample of 22,456 individuals was available for analysis in the present study. The demographic, material and psychosocial characteristics of the effective sample are described in Table 1.

**Table 1:** Demographic, health and material and psychosocial characteristics by intersectional social positions in Spanish adults

		Social class		Gender		Regional development		Intersectional social positions^**a**^							
		non-manual	manual	men	women	high	low	MNH	WNH	MNL	WNL	MMH	WMH	MML	WML
**Total** **(N)**		7891	14,565	11,376	11,080	11,461	10,995	2069	2503	1539	1780	6655	3234	41,113	3563
**Age (mean, range)**		51.97 (20–88)	55.49 (19–88)	54.53 (19–88)	53.97 (20–88)	54.62 (19–88)	53.88 (19–88)	54.12 (21–88)	50.69 (22–88)	53.98 (22–88)	49.54 (20–88)	55.03 (19–88)	57.50 (20–88)	54.51 (19–88)	55.28 (20–88)
**Poor SRH (%)**		17.96	34.99	27.94	30.10	26.76	31.34	17.88	16.26	20.60	18.15	30.26	36.61	33.67	39.88
**Material factors (%)**	*Material scarcity*	1.80	14.42	9.27	10.72	7.72	12.35	1.79	1.60	1.88	2.02	9.90	13.79	15.24	18.69
	*Unstable employ- ment*	6.59	16.87	9.56	17.05	9.05	17.64	2.32	7.39	4.35	12.36	8.81	14.90	15.83	28.12
	*Insecure residen- tial area*	8.97	10.11	9.11	10.33	10.94	8.43	9.18	11.15	6.82	7.53	10.73	12.15	8.49	9.51
**Psycho-social factors (%)**	*Poor social support*	2.43	5.60	4.80	4.16	3.96	5.03	2.61	1.96	2.86	2.53	5.20	4.98	6.27	5.78
	*Lack of social partici- pation*	17.27	42.52	34.89	32.37	29.67	37.79	18.12	14.62	21.12	16.69	38.00	39.30	45.71	46.39

^a^The first letter of the intersectional social position is gender (M = Men, W=Women), the second letter is the social class (M = Manual, N=Non-manual) and the third letter is the regional development (H=High, L = Low)

### Variables

The dependent variable *self-rated health (SRH)* was derived from the following question: Would you say that your overall health is either: very good, good, fair, poor or very poor? The answers were dichotomized into either good health (good or very good coded as 0) or poor health (fair, poor or very poor coded as 1).

SRH is a common measure of an individual’s well-being and health status and has been shown to be a valid and reliable indicator of morbidity and early mortality [36], and that displays social inequalities [37, 38].

The three binary variables of *social positions* were *social class* (manual or non-manual), *gender* (man or woman) and *regional development* (high or low). *Social class* was coded according to the Spanish adaptation of the British Registrar General classification, based on the International Standard Classification of Occupation 2008 [39, 40], with manual class comprising the III-V groups and the non-manual I-III groups of the British Registrar General classification. *Gender* was self-reported in the Living Conditions Survey with two options: woman or man. *Regional development* was derived from the Inequality-adjusted Human Development Index (IHDI) for each Autonomous Community and Autonomous city in Spain in 2010 [41]. Those with the highest IHDI were considered High development regions and those with the lowest IHDI were considered Low development regions.

The three social position variables were combined to form eight *intersectional social positions*: Men Non-manual social class High development regions (MNH); Women Non-manual social class High development regions (WNH); Men Non-manual social class in Low development regions (MNL); Women Non-manual social class in Low development regions (WNL); Men Manual social class in High development regions (MMH); Women Manual social class in High development regions (WMH); Men Manual social class in Low development regions (MML); and Women Manual social class in Low development regions (WML).

Variables potentially reflecting *social processes* underpinning intersectional inequalities in SRH were identified in the Living Conditions Survey. The three material factors *material standards of living, employment conditions,* and *residential environment* and the two psychosocial factors *social support* and *social participation* were selected.

For *material standards of living* the following nine binary items were selected and summed up into an index: having holidays at least 1 week a year away from home; a mobile phone; a television; a computer; access to internet; a washing machine; a car; a private shower, and spending discretionary money weekly on oneself. The index was dichotomised and when four or more items were lacking it was labelled *Material scarcity* [42].

*Employment conditions* was indicated by two items: employment status (wage worker full time, wage worker partial time, self-worker full time, self-worker partial time, student, retired, permanent incapable to work, household worker, other type of economic inactivity), and type of contract (employer, self-employed, permanent wage, temporary wage, and familiar help). *Unstable employment* index was defined when employment status was student, retired, permanent incapable to work, household worker or other type of economic inactivity and when type of contract was temporary wage or familiar help.

*Residential environment* was based on two yes/no questions: existence of delinquency problems and existence of vandalism in the respondent’s residential area. *Insecure residential area* was defined among those with at least one ‘yes’ answer.

*Social support* was based on two yes/no questions: if the respondent had family or friends who they could ask for help and if the respondent had someone to talk to about personal issues. *Poor social support* was defined among those with at least one ‘no’ answer.

*Social participation* was derived from ten items referred to participation in activities the past year such as having: gone to the cinema; gone to the theatre; visited cultural places; gone to sport events; participated in voluntary activities, and participated in political activities; as well as frequency of meeting friends, contacting family members, contacting friends, and participating in social media. *Lack of social participation* was defined as a negative response to seven or more items.

### Statistical analysis

Descriptive statistics comprised frequencies of SRH, age, and explanatory variables across social positions and eight intersectional social positions.

Two different sets of analysis were undertaken to address the aims, all using of multivariate Poisson regressions to estimate prevalence ratios (PR) [43] with SRH as the outcome and social positions as main exposures. The first set of analysis included the three indicators of social positions as mutually adjusted independent variables (corresponding to an additive approach, which does not illustrate the health risks in the intersecting social positions). The second instead utilized the indicator of eight intersectional social positions according to an intersectionality-informed multiplicative approach (which discloses the health risks of multiple intersecting social positions) – with the best-off group (men in non-manual social class from high development regions) as the reference category. For each of the two approaches, four models were created. Model A was adjusted only by age, Model B was adjusted for age and all psychosocial factors, Model C was adjusted for age and material factors, and Model D was adjusted for all factors together. The explained fraction (EF) of each social position and intersectional social position was calculated after every adjusted model versus the crude model given the following equation: ((PR_A-_PR_B_)*100/(PR_A_-1)).

A complete case analysis was conducted when missing data existed, such as for 4580 subjects without classifiable social class as nothing was stated in their occupational status. Out of these, 47% were born 1990–1998 and were therefore students or unemployed young adults. All analyses were carried out with the Stata version 14 statistical package.

## Results

### Socio-demographic characteristics of the samples

The sample analysed in this study comprised of 14,565 adults in the manual class and 7891 in the non-manual class; 11,080 women and 11,376 men; and 11,461 people living in high development regions and 10,995 in low development regions (Table 1).

Out of these, 40% of the respondents in high development regions were non-manual workers while in low development regions 30% were non-manual workers. Gender was homogenously distributed by region, but when it comes to social class men tended to belong to manual class more often that women did (68% v 61%).

### Intersectional inequalities in material and psychosocial disadvantages

**Fig. 1:**
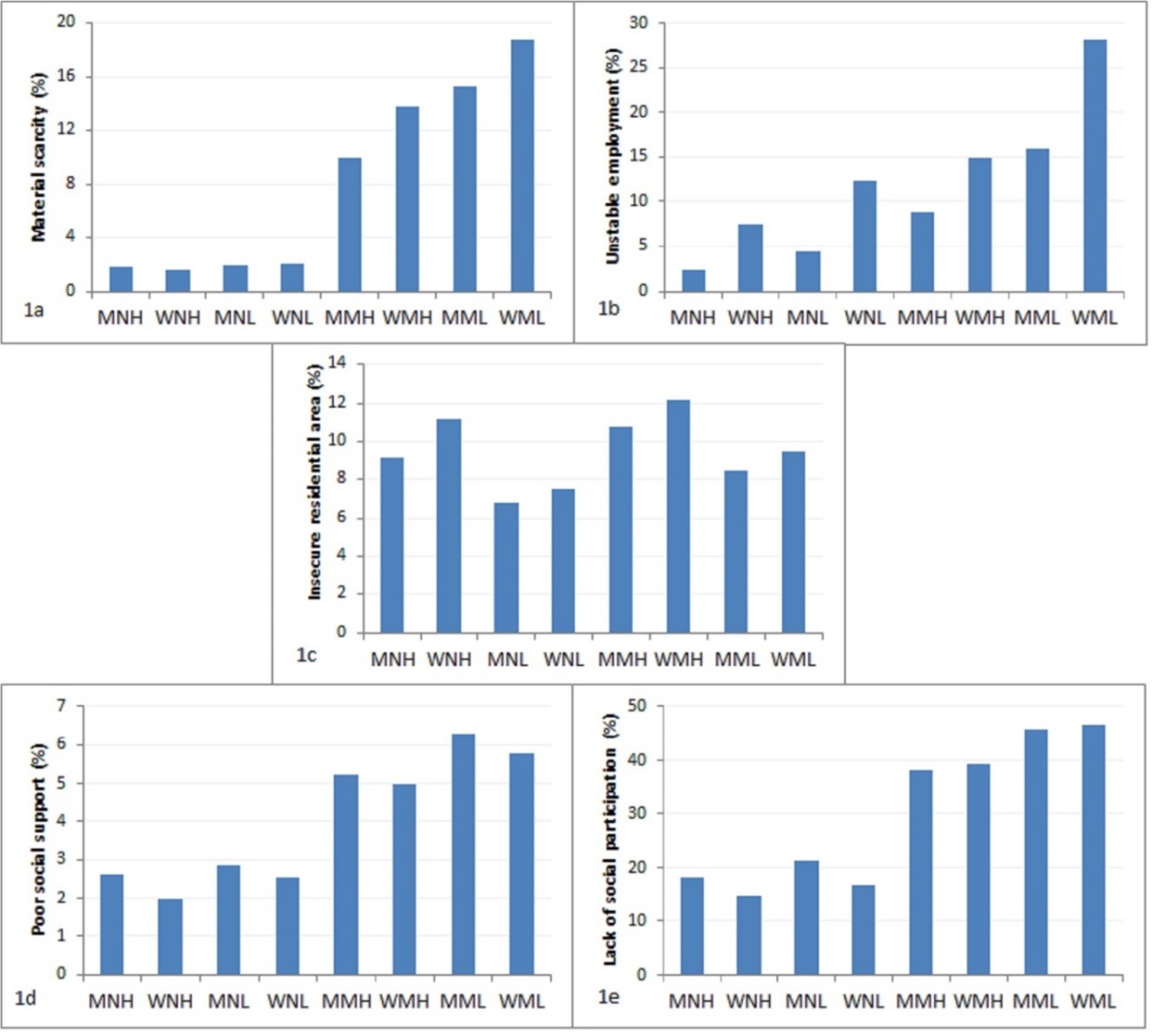
**a-e** Percentage of material and psychosocial factors in eight different intersectional social positions^1^ in Spanish adults. ^1^The first letter of intersectional social position is gender (M = Men, W=Women), the second letter is the social class (M = Manual, N=Non-manual) and the third letter is the regional development (H=High, L = Low)

The distribution of material and psychosocial factors displayed distinctive inequalities between, but also within, the indicators of class, gender and regional development (Table 1 and Fig 1).

Firstly, considering the three indicators one by one, the largest inequalities were found for social class for which manual class consistently displayed more material and psychosocial disadvantages as compared to non-manual social class. There were eight times higher frequency of material scarcity and more than double the frequency of unstable employment, poor social support and lack of social participation, but with similar prevalence of insecure residential area. Women reported unstable employment 80% more often than men, with the other indicators displaying smaller inequalities (< 20% relative difference). The disadvantages for women were material scarcity and insecure residential area and the corresponding for men were social support and social participation. Low development regions reported disadvantages more often (30–90%) than high development regions, except for insecure residential area which was slightly more common in privileged regions.

Secondly, distribution of material and psychosocial factors across intersectional social positions revealed more complex patterns of inequalities not discernible through single indicator inequalities. For example, the triply disadvantaged group (women in manual class from low development regions) reported ten times higher frequency of material scarcity than the triply advantaged group (men in non-manual class from high development regions), which can be compared to the eight times difference between manual and non-manual social classes (Table 1 and Fig. 1a). The triply disadvantaged group also reported twelve times higher frequency of unstable employment than the triply advantaged group, which can compared to the moderate 2–3 times difference between collapsed groups of women and men, manual and non-manual classes, and high and low regional development groups (Table 1 and Fig 1b). This illustrates how the magnitude of the intersectional inequalities cannot be monotonously predicted from single inequalities but depended on life conditions.

The complexity become even more apparent when considering intersectional groups with mixed position of advantage and disadvantage; further illustrating the heterogeneity in life conditions not only between, but also within, the crude categories captured by the single indicators. For example, the intersectional social position with the overall lowest material scarcity was not men but women, in non-manual occupations and high development regions. Moreover, whereas material scarcity, as noted above, was clearly patterned by social class, women in manual class from low development regions reported twofold material scarcity as men in manual class from high development regions (Table 1 and Fig. 1a). Additionally, the small relative advantage of women as a group when it comes to psychosocial resources was restricted only to non-manual class (Table 1, Fig. 1d, e).

### Intersectional inequalities in SRH

**Fig. 2:**
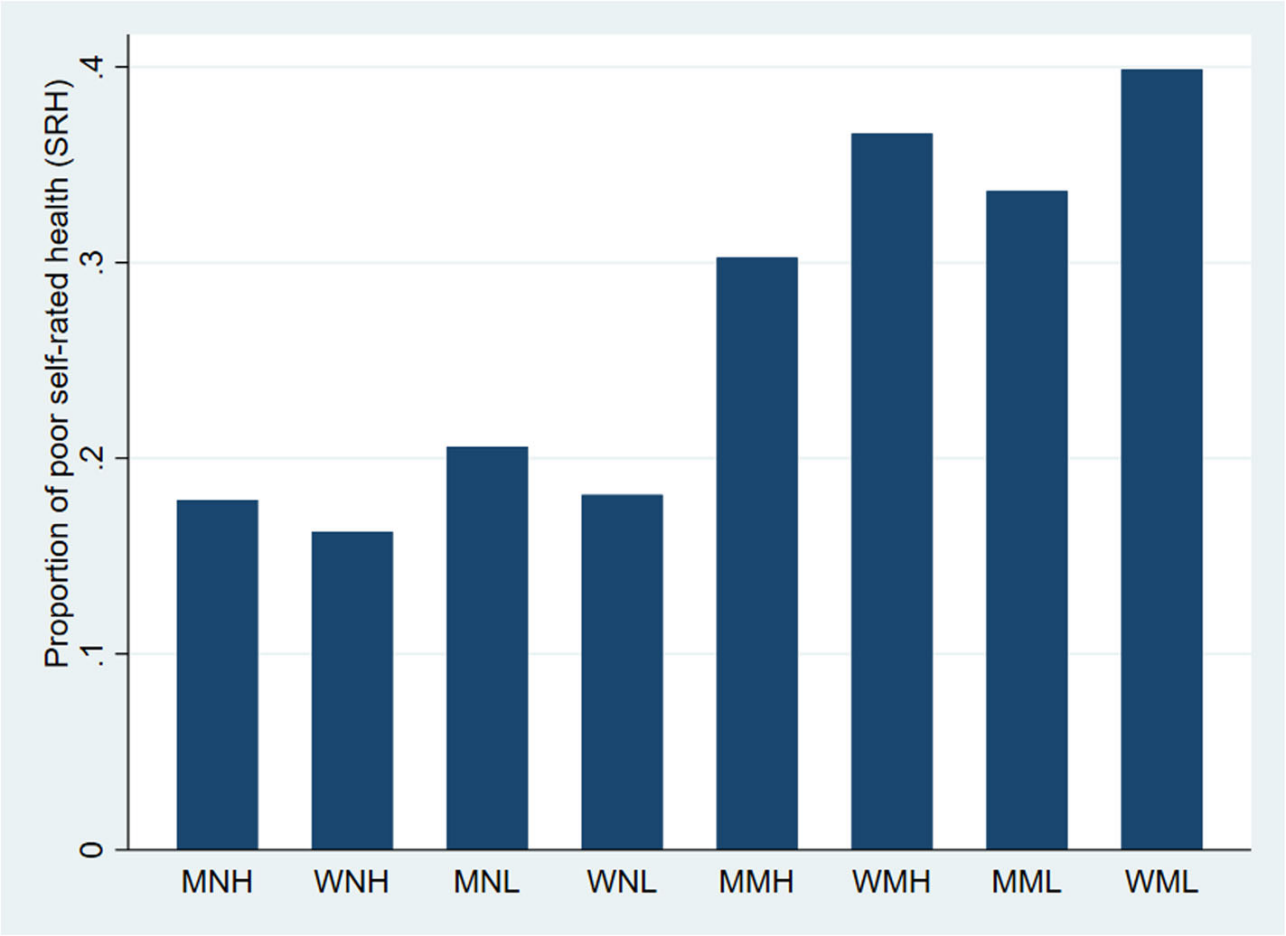
Proportion of poor self-rated health (SRH) in eight different intersectional social positions^1^ in Spanish adults. ^1^The first letter of the intersectional social position is gender (M = Men, W=Women), the second letter is the social class (M = Manual, N=Non-manual) and the third letter is the regional development (H=High, L = Low)

Descriptive patterns indicating complex inequalities between intersectional social positions were also found when it comes to SRH (Fig. 2), and most of these inequalities were also confirmed in age-adjusted Poisson regression models (Table 2). The most advantageous position after age-adjustment was the triply advantaged group of men of non-manual social class from high development regions, while the most disadvantageous position was the triply disadvantaged position of women of manual social class from low development regions, with greater than double prevalence of poor SRH in the latter group (Table 2).

**Table 2:** Age-adjusted prevalence ratios (PR) for poor health: comparisons between intersectional groups in Spanish adults

Exposure group	Reference group							
	MNH	WNH	MNL	WNL	MMH	WMH	MML	WML
	PR	PR	PR	PR	PR	PR	PR	PR
**MNH**	1	0.95	0.86*	0.80**	0.62***	0.56***	0.54***	0.47***
**WNH**	1.05	1	0.90	0.84**	0.65***	0.59***	0.57***	0.50***
**MNL**	1.17*	1.11	1	0.93	0.72***	0.65***	0.64***	0.55***
**WNL**	1.25**	1.19**	1.07	1	0.77***	0.70***	0.68***	0.59***
**MMH**	1.61***	1.54***	1.38***	1.29***	1	0.90**	0.88***	0.76***
**WMH**	1.79***	1.71***	1.54***	1.43***	1.11**	1	0.98	0.85***
**MML**	1.84***	1.75***	1.57***	1.47***	1.14***	1.02	1	0.87***
**WML**	2.11***	2.00***	1.81***	1.69***	1.61***	1.18***	1.15***	1

*p-value < 0.05; **p-value < 0.01; *** *p*-value < 0.001

^1^The first letter of the intersectional social position is gender (M = Men, W=Women), the second letter is the social class (M = Manual, N=Non-manual) and the third letter is the regional development (H=High, L = Low)

While this indicates some degree of cumulative effect of multiple disadvantage, there were also more complex patterns of health within disentangled social positions, contingent on other inequality dimensions. For example, whereas all manual worker intersectional positions displayed higher frequencies of poor SRH than all non-manual positions (Table 2: 29–110% higher prevalence, all *p* < 0.001), manual worker women in low development regions also reported 61% higher prevalence (*p* < 0.001) than their male counterpart in high development regions. Similarly, the same triply disadvantaged group also reported worse health than women in other intersectional positions; 100% higher prevalence of poor SRH than women in non-manual social class and in high development regions (*p* < 0.001); 69% higher prevalence than women in non-manual social class and in low development regions (*p* < 0.001); and 18% higher prevalence than women in manual social class but in high development regions (*p* < 0.01).

The results also illustrate similarities in health risks between certain intersectional groups. For example, while intersectional positions of women overall reported worse SRH than men, women and men in non-manual social class from high or low development regions reported a comparable prevalence of poor SRH (5–7% higher in women, *p* > 0.05).

### The role of material and psychosocial factors according to additive and the multiplicative approaches

Poisson regression analyses were carried out to estimate social inequalities in SRH by social positions of gender, social class and regional development according to the additive and multiplicative approaches, respectively, and to examine the explanatory role of material and psychosocial factors (see Table 3 for analyses according to the additive approach, and Table 4 for analyses according to the multiplicative approach).

**Table 3:** Prevalence ratios (PR) for poor health by social position adjusted for mediators in Spanish adults

Social position	Model A^**a**^		Model B^**a**^			Model C^**a**^			Model D^**a**^		
	Crude model Adjusted by age		All psychosocial factors		PR reduction	All material factors		PR reduction	All factors		PR reduction
	PR	95% CI	PR	95% CI	(%)	PR	95% CI	(%)	PR	95% CI	(%)
*Gender*											
**Men**	1		1			1			1		
**Women**	1.11***	(1.07–1.16)	1.12***	(1.08–1.16)	−4.62	1.07***	(1.03–1.11)	35.93	1.09***	(1.05–1.13)	22.62
*Social class*											
**Non-manual**	1	1	1	1		1	1		1	1	
**Manual**	1.61***	(1.53–1.70)	1.45***	(1.38–1.53)	25.88	1.51***	(1.44–1.59)	16.87	1.40***	(1.33–1.48)	34.31
*Regional development*											
**High**	1	1	1	1		1	1		1	1	
**Low**	1.16***	(1.12–1.21)	1.12***	(1.08–1.16)	26.22	1.13***	(1.09–1.17)	19.29	1.10***	(1.06–1.15)	36.07
***Estimates explanatory factors***											
**Material scarcity**						1.31***	(1.30–1.42)		1.18***	(1.13–1.28)	
**Unstable employment**						1.23***	(1.17–1.32)		1.20***	(1.13–1.26)	
**Insecure residential area**						1.22***	(1.13–1.27)		1.19***	(1.12–1.25)	
**Poor social support**		1.45***		(1.38–1.53)					1.38***	(1.26–1.44)	
**Lack of social participation**		1.47***		(1.44–1.57)					1.42***	(1.38–1.51)	

*p-value < 0.05; **p-value < 0.01; *** *p*-value < 0.001

^a^Model A is the crude model; Model B is adjusted by psychosocial factors; Model C is adjusted by material factors; Model D is adjusted by all factors

**Table 4:** Prevalence ratios (PR) for poor health by intersectional positions^a^ adjusted for mediators in Spanish adults

Intersectional social positions^**a**^	Model A^**b**^		Model B^**c**^			Model C^**d**^			Model D^**e**^		
	Crude adjusted by age		All psychosocial factors		PR reduction	All material factors		PR reduction	All factors		PR reduction
	PR	95% CI	PR	95% CI	(%)	PR	95% CI	(%)	PR	95% CI	(%)
**MNH**	1		1			1			1		
**WNH**	1.05	(0.93–1.19)	1.06	(0.94–1.20)	−24.98	1.03	(0.91–1.16)	41.42	1.05	(0.93–1.18)	9.35
**MNL**	1.17*	(1.03–1.32)	1.14*	(1.00–1.29)	17.17	1.17*	(1.03–1.32)	1.54	1.14*	(1.01–1.29)	16.09
**WNL**	1.25**	(1.09–1.41)	1.24**	(1.10–1.41)	2.73	1.22**	(1.07–1.39)	12.50	1.22**	(1.08–1.39)	11.42
**MMH**	1.61***	(1.42–1.72)	1.47***	(1.33–1.62)	23.85	1.54***	(1.40–1.70)	11.84	1.43***	(1.30–1.58)	29.54
**WMH**	1.79***	(1.58–1.92)	1.64***	(1.49–1.81)	18.89	1.66***	(1.50–1.82)	17.53	1.56***	(1.42–1.72)	29.04
**MML**	1.84***	(1.62–1.96)	1.60***	(1.46–1.76)	27.89	1.70***	(1.54–1.87)	16.62	1.54***	(1.40–1.69)	35.75
**WML**	2.11***	(1.86–2.24)	1.84***	(1.68–2.03)	24.01	1.86***	(1.69–2.05)	22.29	1.71***	(1.55–1.88)	36.24
	***Estimates explanatory factors***										
**Material scarcity**						1.38***	(1.32–1.44)		1.26***	(1.21–1.31)	
**Unstable employment**						1.22***	(1.15–1.29)		1.18***	(1.11–1.24)	
**Insecure residential area**						1.21***	(1.14–1.28)		1.20***	(1.12–1-26)	
**Poor social support**		1.43***		(1.34–1.52)					1.45***	(1.27–1.66)	
**Lack of social participation**		1.49***		(1.43–1.55)					1.45***	(1.39–1.51)	

*p-value < 0.05; **p-value < 0.01; *** *p*-value < 0.001

^a^The first letter of the intersectional social position is gender (M = Men, W=Women), the second letter is the social class (M = Manual, N=Non-manual) and the third letter is the regional development (H=High, L = Low)

^b^Model A is the crude model

^c^Model B is adjusted by psychosocial factors

^d^Model C is adjusted by material factors

^e^Model D is adjusted by all factors

The additive approach revealed that social class was the inequality dimension with the most remarkable health inequalities, amounting to 61% higher prevalence of poor SRH among manual compared to non-manual social class (Model A, Table 3). Smaller but significant inequalities were also found for gender and regional development. As indicated by the explained fraction (EF), psychosocial and material factors partially, but not completely, explained these inequalities. Psychosocial factors (Model B) explained about a fourth of the large class inequalities (EF = 26%) and the smaller regional inequalities (EF = 26%) in SRH but did not contribute to the explanation of the gender inequalities (EF = -5%). Material factors (Model C), had a greater relative importance for gender (EF = 36%) than social class (EF = 17%) or regional (EF = 19%) inequalities. As a result, all factors together (Model D) explained a larger portion of social class (EF = 34%) and regional inequalities (EF = 36%) but less of gender inequalities (EF = 23%). All inequality estimates remained statistically significant (*p* < 0.001) even after full adjustment.

To analyse the inequalities in a comparable manner but according to a contrasting multiplicative approach, the best-off intersectional position after adjusting by age (the triply advantaged group men in non-manual social class in high development regions) was used as reference category (Table 4). As noted above, in contrast to the additive approach, these analyses illustrated both heterogeneity within social positions as well as cumulative effects of multiple disadvantages. For example, whereas the additive approach estimated a 61% higher prevalence of poor SRH between social classes (Model A, Table 3), the multiplicative approach revealed inequalities of a similar magnitude even within manual social class positions, contingent on the other inequality dimensions, and inequalities amounting to 111% higher prevalence of poor SRH between the triply advantaged and the triply disadvantaged groups (Model A, Table 4).

Discrepancies between the additive and multiplicative approach were also evident when the indicators of social processes where taken into account. Overall, psychosocial factors explained inequalities mostly involving those intersectional social positions which had a higher relative frequency in both psychosocial factors (Model B, Table 4: EF_WML_ = 24%; EF_MML_ = 28%; EF_MMH_ = 24%). Specifically, the slightly increased gender inequality when taking psychosocial factors into account in the additive approach (Model B, Table 3: EF = -5%) was only evident for women in non-manual social class from high development regions (Model B, Table 4: EF_WNH_ = -25%), while psychosocial factors in contrast explained a considerable portion of the inequalities involving the triply disadvantaged women (EF_WML_ = 24%). Moreover, the sizeable explanation by psychosocial indicator of social class (EF = 26%) and regional (EF = 26%) inequalities in the additive approach (Table 3) were comparable only for the specific intersectional position of men in manual social class from low development regions in the multiplicative approach (Table 4: EF_MML_ = 28%).

Whereas material factors explained a large portion of the overall gender inequalities (Model C, Table 3: EF = 36%), the multiplicative approach showed their importance also differed markedly for intersectional social positions within the same gender; from 12.5 to 21.4% for women and from 1.5 to 16.6% for men (Model C, Table 4). A similar variation in explanatory power reflecting the intersectional inequalities was seen when adjusting for material factors: PR for non-manual class groups ranged from 1.03 to 1.22 while PR for manual class groups ranged from 1.54 to 1.86.

The full model involved the greatest explained fractions for all intersectional positions except for women of non-manual class (Model D, Table 4), which instead were better explained by material factors only (Model C: EF_WNH_ = 41%; EF_WNL_ = 12%), and for men from non-manual class and low development regions which were better explained by psychological factors only (Model B: EF_MNL_ = 17%). Among all intersectional inequalities, the PR of manual class positions decreased the most when adjusting by all factors. However, the considerable explained fractions for social class (EF = 34%) and regional (EF = 36%) inequalities in the additive approach (Model D, Table 3) were only seen for manual social class from low development regions in the multiplicative approach (Model D, Table 4: EF_MML_ = 36%; EF_WML_ = 36%).

## Discussion

This cross-sectional study on Spanish adults is the first of its kind to investigate how intersectional social positions of gender, social class and regional development are reflected in population patterns of SRH, and to examine the contributions of the intermediary social processes material and psychosocial factors to inequalities in SRH.

Overall, the results of the intersectionality-informed multiplicative analysis pointed towards cumulative albeit not monotonous health effects of multiple disadvantages, which corresponds to findings of others [11]. The findings also illustrate how social categories, which conventionally are treated as homogenous in additive analytical approaches, in fact can be highly heterogeneous when it comes to their health risks. Taking the example of gender and the social category of women, women in non-manual occupation and from high development regions reported health comparable to their male counterpart. In contrast, women who instead worked in manual occupation and lived in low development regions reported twice the frequency of poor health. Moreover, the role of material and particularly psychosocial factors varied considerably to explain gender-related inequalities when taking into account the intersections with social class and regional development. Similar heterogeneous results were found within social classes. These findings thereby challenge conventional notions, additive analytical approaches and policies that consider women or blue-collar workers as homogenous groups with worse health than the equally homogenous social categories of man or white-collar employees.

When it comes to the role of the explanatory factors, Malmusi [26] suggested that individual income contributes importantly to gender inequalities in health. We indeed found that unstable employment contributed substantially to gender inequalities in SRH, especially when considering intersectional social positions, a finding that was underpinned by the ubiquitous material disadvantage in the triply disadvantaged group. For example, although material scarcity was clearly patterned by social class, women in manual class in low development regions reported twice as much material scarcity as men in manual class in high development regions did. This emphasizes how inequity in access to material resources plays a widespread role for social inequalities in Spain.

Several studies [6, 44, 45] have noted that social class inequalities in SRH are related to material factors, such as employment situation and material standards of living. In the present study psychosocial factors explained inequalities to a slightly greater degree than material factors for all manual social class groups, especially those from low development regions. As Iyer [46] mentioned, when social positions interact with each other the consequences (e.g. in terms of health or social determinants of health) are not necessarily uniform, but instead contingent on the particular setting under study. It is possible that the context and the set of indicators of the present study may have caused this high explanatory value of psychosocial factors among manual social class groups.

Moreover, inequalities within social positions were explained by intermediary factors in a different manner than inequalities in intersectional social positions. For example, while psychosocial factors did not contribute to the small gender inequalities in SRH in the additive analyses, the multiplicative analyses revealed that the importance of psychosocial factors for gender-related inequalities in SRH where highly contingent on social class and regional development. As Bauer [9] have remarked, intersectional multiplicity is necessary to not only describe the nuances of population patterns of health, but also to understand how social processes heterogeneously affect intersectional social positions, thereby creating diverse experiences of privilege or discrimination and ultimately complex patterns of population health. The results of the present study illustrate some of this complexity.

### Strengths and limitations

One of the study’s strengths is the large and rich random population-based sample with rather high participation rate that allows creating intersectional categories with enough statistical power. However, the cross-sectional design limits causal inference. Selection bias might be present since 47% of the participants excluded because of their lack of occupational classification were born between 1990 and 1998.

The fact that the study is based on self-reported survey data may introduce response and common-method bias. Socio-economic context was assessed through Inequality-adjusted Human Development Index. Other measures that shed the light on oppressing processes could be used in further studies such as: expenditure and allocation of Basic Public Services or the manner each Autonomous Community applies health related laws (such as Royal Decree Law 16/2012).

The measures used in the present study are all used extensively in population surveys in high-income countries, but with some exceptions (e.g. SRH) the majority of them have not been subjected to validation procedures. Lack of reliability and validity of the measures could bias the reported findings, for example bias introduced due the relatively high proportion of proxy interviews in the survey, as noted by Eurostat [27].

Any inference about the relative and joint importance of sets of explanatory factors, such as material and psychosocial factors, ultimately depend on the specific set of factors included in the analysis. There may be many other factors that could potentially explain SRH inequalities but which were not recorded in the Living Conditions Survey [27]. For instance, other material conditions such as access to service provision or housing conditions; or other psychosocial indicators such as work demands, or negative life events are missing. Likewise, lifestyle (e.g. smoking, alcohol consumption or physical activity) are lacking.

## Conclusions and implications for policy

Using an intersectionality-informed analytical approach, this study illustrates the pervasiveness and entanglement of social inequalities in SRH health in Spain. The results reinforce the notion that different axes of inequality are intertwined and are expressed in complex population patterns of health, which in turn are underpinned by complex social inequities in access to material and psychosocial resources. In order to show power structures that may influence SRH, addressing multi-level interactions may therefore necessary. Deeper understanding of Spain’s public policies and institutional structures is needed in order to disentangle mechanisms underlying social and related health inequalities, where the contingency of social positions need to be considered. Local and national policies are needed, particularly for women in manual social class, in order to improve employment conditions such as access to decent jobs, salaries over the minimum wage, stable working conditions and availability of unemployment benefits in the least developed regions of Spain; as well as creation of health promoting spaces were social participation is encouraged. More intersectionality-informed studies are required to provide evidence to inform policies that can promote health equity in the Spanish population.

## Data Availability

The datasets analysed during the current study are available in the INEbase repository of Instituto Nacional de Estadística (National Institute of Statistics): https://www.ine.es/dyngs/INEbase/es/operacion.htm?c=Estadistica_C&cid=1254736176807&menu=resultados&secc=1254736195153&idp=1254735976608

## Abbreviations

EF: Explained Fraction
IHDI: Inequality-adjusted Human Development Index
MMH: Men Manual social class High development regions
MML: Men Manual social class in Low development region
MNH: Men Manual social class in High development region
MNL: Men Non-manual social class in Low development regions
PR: Prevalence Ratio
SRH: Self-Rated Health
WMH: Women Manual social class in High development regions
WML: Women Manual social class in Low development regions
WNH: Women Non-manual social class High development regions
WNL: Women Non-manual social class in Low development region
